# Median quartet tree search algorithms using optimal subtree prune and regraft

**DOI:** 10.1186/s13015-024-00257-3

**Published:** 2024-03-13

**Authors:** Shayesteh Arasti, Siavash Mirarab

**Affiliations:** 1 Computer Science and Engineering Department, University of California, San Diego, 9500 Gilman Drive, La Jolla, CA 92093 USA; 2 Electrical and Computer Engineering Department, University of California, San Diego, 9500 Gilman Drive, La Jolla, CA 92093 USA

**Keywords:** Phylogenetics, Gene tree discordance, Quartet score, Quartet distance, Subtree prune and regraft, Tree search, ASTRAL

## Abstract

Gene trees can be different from the species tree due to biological processes and inference errors. One way to obtain a species tree is to find one that maximizes some measure of similarity to a set of gene trees. The number of shared quartets between a potential species tree and gene trees provides a statistically justifiable score; if maximized properly, it could result in a statistically consistent estimator of the species tree under several statistical models of discordance. However, finding the median quartet score tree, one that maximizes this score, is NP-Hard, motivating several existing heuristic algorithms. These heuristics do not follow the hill-climbing paradigm used extensively in phylogenetics. In this paper, we make theoretical contributions that enable an efficient hill-climbing approach. Specifically, we show that a subtree of size *m* can be placed optimally on a tree of size *n* in quasi-linear time with respect to *n* and (almost) independently of *m*. This result enables us to perform subtree prune and regraft (SPR) rearrangements as part of a hill-climbing search. We show that this approach can slightly improve upon the results of widely-used methods such as ASTRAL in terms of the optimization score but not necessarily accuracy.

**Supplementary Information:**

The online version contains supplementary material available at 10.1186/s13015-024-00257-3.

## Introduction

The NP-Hard [1] problem of finding a tree that minimizes the total quartet distance to a set of given trees has found wide-ranging applications in recent years [2]. The quartet distance between two unrooted trees is obtained by dividing each tree into all its quartets (choices of four taxa) and counting quartet topologies that do not match [3]. While studying this* quartet median tree problem* is not new [4, 5], its renewed popularity is a result of its connection to a broader trend in phylogenomics – the embrace of methods that account for discordance between gene trees and species trees [6]. While various approaches exist for accounting for such discordance when inferring a species tree [7], many of these methods rely on quartets. There is a reason for the use of quartets. As originally noted by Allman et al. [8] for the multi-species coalescent model (MSC) [9, 10] of incomplete lineage sorting (ILS) and later extended to models of duplication and loss (GDL) [11, 12], HGT [13], and even ILS+GDL [14], on a quartet species tree, the unrooted gene tree topology matching the species tree has a higher probability of being observed than the two alternative topologies. Some methods (e.g., ASTRAL [15]) have used this observation to directly use the median quartet tree problem as a way of estimating species trees. Others (e.g., [16–18]) have used this observation to infer individual quartet species trees using some criterion and then combine them. Either way, taxa are divided into quartets.

Dividing *n* taxa naively into quartets will require $\Omega (n^4)$ time just to list the quartets, making any resulting algorithm impractical on large datasets. Some methods still use this approach but subsample quartets to an asymptotically smaller size (e.g., a quadratic or even $O(n\log (n))$ number [19]). However, subsampling can be avoided while achieving high scalability using improved data structures and algorithms. For the simplest problem of computing the quartet distance between two trees, which naively would require $\Omega (n^4)$ time, a straightforward algorithm can achieve quadratic time [20]. This requires the post-traversal of one tree and comparing each node versus each node of the other tree, keeping track of the number of shared children below these nodes. This approach has been at the heart of ASTRAL since version 2 [21]. If we allow ourselves to use much more sophisticated data structures, we can do even better. Brodal et al. [22] have designed a complex data structure called Hierarchical Decomposition Tree (HDT), which, along with a host of counters and other algorithmic tricks, enable a $O(n \log ^2(n))$ algorithm for computing quartet distance. Mai and Mirarab [23] later extended this approach to solve the problem of adding one taxon to a tree so that the updated tree has the minimum possible distance to a set of *k* input trees. Zhang and Mirarab [24] used similar ideas to infer a tree by successively adding one taxon to a growing tree in a tool called ASTER. Thus, quadratic and sub-quadratic quartet methods without subsampling are widely available.

Available quartet-based median tree inference methods differ from most other phylogenetic inference methods in their approach. ASTRAL [15], which is perhaps the most widely used method for this problem, uses a dynamic programming algorithm (an approach with long history; see [25–27]) to solve the problem exactly in exponential time or under some constraints in polynomial time ($O((nk)^{2.726})$ in the worst case and close to $n^2k^2$ empirically [28]). The ASTER package [24, 29] uses several rounds of step-wide addition with random orders of adding taxa, followed by a dynamic programming step similar to ASTRAL to combine these greedy results. Earlier methods such as wQMC use graph-based techniques [30]. Thus, none of these methods use the hill-climbing search algorithms used by most other phylogenetic inference tools. While ASTRAL has been scalable, it is not clear if the reason is the use of a constrained dynamic programming algorithm or if an efficient hill-climbing could be as efficient or perhaps even more. If the improvements are due to constrained dynamic programming, perhaps we should explore similar methods for other problems. On the other hand, it is possible that hill climbing can improve quartet-based estimation in terms of running time, accuracy, or both.

Hill climbing tree search requires efficient methods of updating the score after a rearrangement. This is often straightforward for Nearest Neighbour Interchange (NNI) moves around the current tree *T*. However, many modern methods use Subtree Prune and Regraft (SPR) rearrangements in addition to NNI. SPR rearrangement is defined on an edge $(u,u')$, selecting one end, say *u*, as the pruning point. The $(u,u')$ edge is pruned at *u* and is grafted back on an edge $(v,v')$ by creating new edges (*v*, *u*) and $(u,v')$. Assume we know the quartet distance of a tree to another tree before an SPR move. How should we update the distance after the move? We could use the Brodal et al. [22] (called B13 hereafter) method and simply recompute the score in $O(n \log ^2(n))$ time. Doing so, we would need $O(n^2 \log ^2(n))$ time to find the optimal SPR move for a given $(u,u')$; one “round” of SPR would in the worst case require trying pruning all edges of a tree, which would need $O(n^3 \log ^2(n))$. Thus, a single SPR round can start to become infeasible, and we need many rounds.

Our goal in this paper is to enable SPR-based hill climbing for the quartet median tree given a set of *k* input trees. Mai and Mirarab [23] extended the B13 algorithm to optimally add a single taxon to a tree in $O(n \log ^2(n) k)$ time. For a pruned subtree of size *m*, we can repeatedly use this algorithm to find the optimal grafting destination in $O((n-m)m \log (n-m) \log (n) k)=O(n^2 \log ^2(n) k)$ time. In this paper, we show that we can do even better: In $O((n-m) \log (n-m) \log (n) k)$ time, we can find the optimal position for the pruned subtree of size *m*. Surprisingly, this time does not increase with *m* and is only $O(n \log ^2(n) k)$ in the worst case when *m* and *n* are of the same order. This worst-case for finding the optimal grafting position is surprisingly the same as the time needed for computing the quartet score. With this algorithm, a full SPR round requires only $O(n^2 \log ^2(n) k)$ time because *O*(*n*) SPR sources need to be tested.

Our theoretical results enable us to design a hill-climbing algorithm for the median quartet tree problem. We built such a tool, called Q-SPR. In simulation and on real data, we show that starting from ASTRAL-III trees, SPR moves can improve the quartet score marginally; however, these improvements do not result in meaningful improvements in accuracy. Starting from a tree built using a stepwise addition performed using tripVote leads to a complete hill-climbing software, which, while competitive with ASTRAL-III in terms of accuracy, is substantially slower in practice under the conditions we tested here. Our results indicate that the dynamic programming strategy of ASTRAL is indeed beneficial for achieving fast running time. However, we note that Q-SPR still is useful for further refining ASTRAL-III output. Moreover, its memory and running time depend on *k* only linearly, which is better than ASTRAL-III, which depends on *k* super-quadratically, for handling tens or hundreds of thousands of genes.

## Materials and methods

### Notations

We denote a tree by $T=(V_T,E_T)$ and let $L_{T} \subseteq V_T$ be the leaftset. An edge $(v,u)\in E_T$ is directed from the parent *v* to the child *u*. We refer to the root of a tree *T* by $r_{T}$ and use $d_{T}$ to denote its maximum node degree, omitting the subscript when clear. We use $u^{\uparrow }$ to denote the parent of a node $u {\in V_T {\setminus } \{r_{T}\}}$. We let $L_{u}$ denote the set of leaves below *u*. Removing the edge $(u^{\uparrow }, u)$ from the tree *T* creates two subtrees, one with and one without the vertex *u*, denoted by ${T}^\vee _{u}$ and ${T}^\wedge _{u}$, respectively. Note that any resulting degree-2 node in ${T}^\wedge _{u}$ is suppressed, connecting its child to its parent. We use ${T} \overset{u}{\circ }T'$ to denote the *placement* of a rooted subtree $T'$ on the edge $(u^{\uparrow }, u)$ of *T*: we divide $(u^{\uparrow }, u)$ to $(u^{\uparrow }, v)$ and (*v*, *u*) by adding *v* and then connect $T'$ to *v* by adding the edge $(v, r_{T'})$. When *u* is the root $r_{T}$, we add a new root $r^*$ and create two new edges $(r^*, u)$ and $(r^*, r_{T'})$. The tree *T* can be rerooted at an edge $e=(v, u)$ to obtain the rerooted tree $T^\oplus _{u}$; to do so, we divide *e* into (*v, r*) and (*r*, *u*) where *r* is the new root and reverse the direction of all edges in the path from *r* to $r_T$. Finally, we remove all degree-2 nodes other than the new root by connecting their children to their parents in the rerooted tree $T^\oplus _{u}$.

**Fig. 1:**
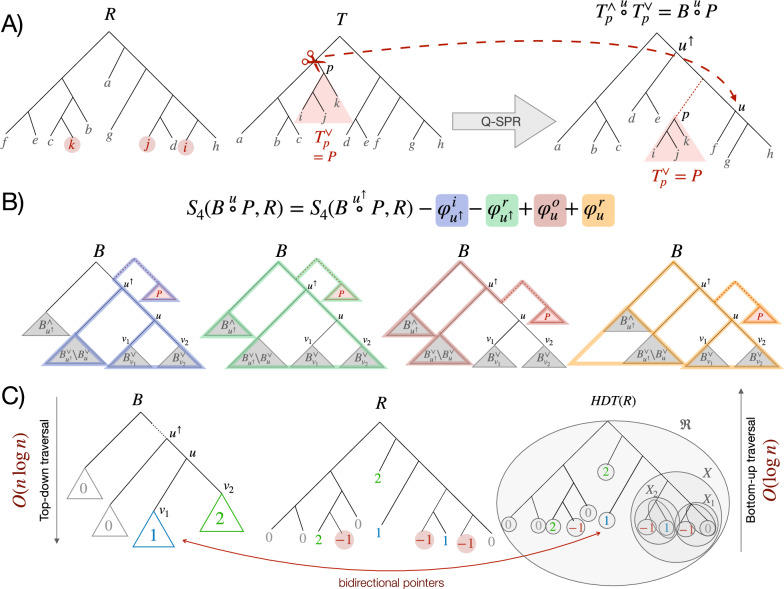
An overview of the Q-SPR problem and the algorithm. **A** The query tree *T*, a set of reference trees $\mathcal {R}$ (one shown), and a node $p$ are given. An SPR move removes a subtree ${T}^\vee _{p}$ ($P$ for short) and places it somewhere on the rest of the *T*, denoted by ${T}^\wedge _{p}$ ($B$ for short). The Q-SPR problem seeks the placement above node *u* denoted by ${{T}^\wedge _{p}} \overset{u}{\circ }{T}^\vee _{p}$ (or ${B} \overset{u}{\circ }P$ for short) with the maximum number of shared *unrooted* quartets with the reference tree(s). **B** The recursive equation of Lemma 1: each of the counters corresponds to a certain type of solo quartet, color-coded here. Subtracted counters are to fix double-counting by other counters, as shown in Table 3. **C** Reference tree *R* is represented as an HDT. For each node *u* of $B$, the leaves are colored such that they correspond to the sides of that node. Outside *u* is colored 0, the larger child is colored 1, and the rest are colored $2\ldots d_u$. The recoloring of $B$ results in the recoloring of the HDT nodes representing *R*. Note that query nodes are always colored $-1$ and are never recolored. Overall, we need $O(n \log (n))$ leaf recoloring during the top-down traversal of $B$, each of which can require a $O(\log (n))$ bottom-up traversal of the HDT

A tree *T* can be restricted to any arbitrary set of three leaves in $L_{T}$ (suppressing nodes with a single child in the process) [31]; we call each of those a triplet of *T*. We call the least common ancestor (LCA) of any three leaves in *T* its *anchor*. We similarly define a *rooted quartet* and its anchor by restricting *T* to any set of four leaves. Unrooting this tree gives us the *unrooted quartet*; when not specified, the term *quartet* refers to the unrooted case. For a triplet of leaves $\{x, y, z\}$, we say $T_1$ and $T_2$ match, or the triplet is *matching* or *shared* between $T_1$ and $T_2$, iff $\{x, y, z\} \subseteq L_{T_1} \cap L_{T_2}$, and $T_1$ and $T_2$ have the same triplet topologies when restricted to $\{x, y, z\}$. Similarly, a quartet of leaves $\{w, x, y, z\}$ is called a matching or shared quartet of $T_1$ and $T_2$ iff $\{w, x, y, z\} \subseteq L_{T_1} \cap L_{T_2}$, and the restricted trees have the same *unrooted* topology in both trees.

### Problem definition

We use $S_3(T_1, T_2)$ to denote the number of triplets that match between the two trees and use $S_4(T_1, T_2)$ to denote the number of quartets matching between two trees.

#### **Definition 1**

(Q-SPR Problem) Given a rooted query tree $T=(V_T,E_T)$, a set of arbitrarily rooted reference trees $\mathcal {R} = \{R_1, R_2,..., R_k \}$, and $p\in V_T {\setminus \{r_{T}\}}$, find

$$\begin{aligned} u^* = \mathop {\mathrm {arg\,max}}\limits _{u\in V_{{T}^\wedge _{p}}} \sum _{i=1}^{k} S_4({{T}^\wedge _{p}} \overset{u}{\circ }{T}^\vee _{p}, R_i) \end{aligned}$$

where $u^*$ is the optimal placement of the pruned subtree ${T}^\vee _{p}$ and ${{T}^\wedge _{p}} \overset{u^*}{\circ }{T}^\vee _{p}$ gives the optimal output (Fig. 1A). Let $n = |L_{T}|$ and $m = |L_{p}|$, and thus $|L_{{T}^\wedge _{p}}| = n-m$.

The Maximum-matching Quartet Placement (MQP) problem of Mai and Mirarab [23] is a special case of Q-SPR where $m=1$ and a single query taxon *q* is *placed* on a given tree. They also defined a Maximum Triplet Rooting (MTR) problem where the goal is to root a given tree *T* based on a set of rooted reference trees $\mathcal {R}$ so that the rooted tree $T^*$ maximizes $\sum _{i = 1}^{k} S_3(T^*,R_i)$. MQP and MTR turn out to be equivalent: All reference trees can be rooted at *q*, and *q* can be placed at the root of the output of MTR to solve MQP.

### Background: tripVote algorithm

Our solution to Q-SPR builds on the tripVote [23] algorithm that solves MTR and MQP using an extension of the B13 algorithm. Recall tripVote seeks to optimally root a query tree *T* with respect to a reference tree *R*. tripVote is based on a recursion:

1 $$\begin{aligned} S_3(T^\oplus _{u}, R) = S_3(T^\oplus _{u^{\uparrow }}, R) - \tau _{u^{\uparrow }}^i - \tau _{u^{\uparrow }}^r + \tau _u^o + \tau _u^r \end{aligned}$$

where *R* is a single reference tree *R*, and the $\tau$ values are counters defined in Table 1. Effectively, this recursion computes the score of each rerooting $T^\oplus _{u}$ of *T* based on precomputed counters and the score of the rerooting on the parent of the current node. The $\tau _u^i$, $\tau _u^o$, and $\tau _u^r$ counters are computed in a top-down traversal of *T*, and Equation (1) makes it trivial to find the optimal rooting once these counters are computed.

**Table 1:** Each counter gives the number of triplets shared between a query tree *T* (or a rerooting of *T*; 2nd column) that are anchored at a particular node of *T* (e.g., *u*; 3rd column) and a component *X* of the HDT (4th column)

	Rerooting *T* at	Shared triplets anchored at	Under HDT component
Counters of a node *u* of *T*			
$\tau _{u}^i$	no rerooting	node *u*	$\mathfrak {R}$
$\tau _{u}^r$	$(u^{\uparrow },u)$	new root of $T^\oplus _{u}$	$\mathfrak {R}$
$\tau _u^o$	$(u^{\uparrow },u)$	new sister to *u* (old $u^{\uparrow }$)	$\mathfrak {R}$
Counters of a component *X* of the HDT			
$\pi ^X_0$	no rerooting	node *u*	*X*
$\rho ^X$	$(u^{\uparrow },u)$	new root of $T^\oplus _{u}$	*X*
$\pi ^X_j$	$(u,v_j)$	new sister to $v_j$ (old *u*)	*X*

HDT represents the reference tree *R* and is rooted at $\mathfrak {R}$

*Coloring scheme.* To compute the counters, tripVote follows the coloring scheme of B13. As it traverses *T* in preorder, on visiting a node *u*, it recolors the leaves so that each side of *u* has a different color. Thus, we need $d_T$ colors, which we refer to with an index in $[0,d_T-1]$. Around a node *u* of *T* with degree *d* and children ${v_1, \ldots , v_{{d-1}}}$ and parent $u^{\uparrow }$, we can obtain *d* subtrees: ${T}^\vee _{v_1}, \ldots , {T}^\vee _{v_{d-1}}$, and ${T}^\wedge _{u}$. After recoloring, the leaves in ${T}^\wedge _{u}$ are colored 0, and the leaves in the subtrees ${T}^\vee _{v_1}, \ldots , {T}^\vee _{v_{d-1}}$ are colored $1, \ldots , d-1$, respectively. Colors are marked on leaves of both *T* and *R*, which are linked using bidirectional pointers. Conceptually, the node *u* partitions leaves of *T* into *d* parts, and leaves of each part can be located anywhere in the tree *R* (i.e., do not necessarily form a clade). The coloring of a leaf in *R* helps us track which part (i.e., side of *u* in *T*) it belongs to. Colors are updated one leaf at a time. After all leaves are colored to match *u*, we traverse *R* from recolored leaves to the root and update a set of counters kept at each node of *R*. These counters are functions of the colors of the leaves in *R* and are used in counting the number of shared triplets between *T* and *R* that match certain criteria specific to node *u*, given in Table 1.

*Hierarchical Decomposition Tree (HDT).* To make recoloring fast, B13 represent *R* using a data structure called HDT (Hierarchical Decomposition Tree). HDT is a locally balanced tree that is built on a rooted tree *R* with *n* nodes in *O*(*n*). Each node (also called component) of the HDT corresponds to a set of connected nodes in *R*. There are four possible types of components in an HDT listed in Table 2. An HDT is constructed in an iterative process. Initially, every leaf and internal node of *R* is represented with an *L* or *I* type component in the HDT, respectively. During each round of construction, two components are merged to form a new HDT component, which is their parent (i.e., *includes* them). The type of the new node depends on what types are merged (Table 2). The order of these merge operations is specified by B13. The process stops when we build the root component of the HDT ($\mathfrak {R}$), which corresponds to the set of all nodes of *R*.

Once the HDT is built, its nodes are assigned counters that keep getting updated. Each component *X* of the HDT has two main counters $\rho ^X$ and $\pi ^X_j$, defined in Table 1 (of these, $\rho ^X$ is similar to B13 counters and $\pi ^X_j$ is specific to tripVote). Note that these counters keep changing value as we traverse *T and recolor its leaves*. After we visit a node *u* of *T*, leaf recoloring and counter updates are triggered; after this is done, counters of the HDT ($\rho ^X$ and $\pi ^X_j$) have values defined in Table 1 specific to *u*. Each HDT counter of a component *X* is the sum over all its children, plus extra triplets counted specifically at *X*. Due to this cumulative nature and based on their definition (Table 1), $\rho ^X$ and $\pi ^X_j$ at the root of the HDT ($\mathfrak {R}$) give $\tau _u^i$, $\tau _u^o$, and $\tau _u^r$ values of *T*; that is, $\tau _u^i = \pi ^\mathfrak {R}_0$, $\tau _u^r = \rho ^\mathfrak {R}$, and $\tau _{v_j}^o = \pi ^\mathfrak {R}_j$. To recolor each leaf of the HDT, we update $\rho ^X$ and $\pi ^X_j$ counters for all its ancestors; at the root $\mathfrak {R}$, we can update the corresponding $\tau _u^i$, $\tau _u^o$, and $\tau _u^r$ counters of *T*. The $\rho ^X$ and $\pi ^X_j$ counters, in turn, are based on $O(d^2)$ other auxiliary counters kept for each node of the HDT and more complex recursive equations to update these counters (we revisit these recursions later). These updates can be done  with a time complexity that, per node, does not depend on *n*, requiring $O(d^2)$ amortized over all calculations.

**Table 2:** HDT components types

Type	Rule	Description
*I*	initial	Corresponds to an internal node in *R*. Every *I* component is a leaf in the HDT.
*L*	initial	Corresponds to a leaf node in *R*. Every *L* component is a leaf of the HDT and is considered a *C* type as well.
*G*		Corresponds to a subtree of *R* (i.e., every node of *R* descendent from any node in a *G* is also in that component.)
	$GG\rightarrow G$	Two *G* components can merge if their roots are siblings in *R*.
	$C\rightarrow G$	A *C* can convert to a *G* if it corresponds to a subtree of *R*.
*C*		Corresponds to a connected subset of nodes in *R*. The children of a *C* satisfy this: there exists an *R* node in one child that is the ancestor of an *R* node in the other child.
	$IG\rightarrow C$	An *I* and a *G* component can merge when the root of *G* is a child of the internal node in *R* represented by *I*.
	$CC\rightarrow C$	Two *C* components can merge if the root of one in *R* is the ancestor of the other in *R* and they form a connected subset of nodes.

Each component in the HDT corresponds to a set of connected nodes in the reference tree *R*, and can have four types. To construct HDT, B13 follows the rules provided here to compose new components and transform existing ones. B13 show that these rules applied with the appropriate order result in a locally balanced hierarchy.

*Running time.* In terms of the running time, recoloring one node of the HDT requires only $O(d_T^2\log (n))$ calculations because of the local balance property of the HDT (i.e., each component of the HDT with *m* leaves has $O(\log (m))$ height). Also, as we traverse *T*, using a smaller-half trick adopted from B13, we perform at most $O(n\log (n))$ leaf recoloring on the HDT (a naive recoloring scheme would require $O(n^2)$ recolorings). Since each counter is updated in constant time, the total recoloring time is $O(d_T^2 n \log ^2(n))$ for one reference tree. If we have *k* reference tree, we process them independently, obtaining $O(k d_T^2 n \log ^2(n))$. Note that the HDT structure can be constructed from *R* in *O*(*n*) time.

### Optimal quartet SPR placement in sub-quadratic time

For ease of notation, we let $B$ denote the backbone tree after pruning (i.e., ${T}^\wedge _{p}$), let $P$ denote the pruned subtree (i.e., ${T}^\vee _{p}$), and let $d=d_T$. First, we note:

#### Observation 1

To find the number of shared quartets between any potential placement of $P$ on $B$ and the reference tree *R*, we only need to count those quartets that have a single leaf from ${P}$ and three leaves from ${B}$.

#### Proof

The topologies for quartets with zero or more than one leaves from ${P}$ do not depend on the placement of $P$. Those with zero or four from ${P}$ clearly have no relation to its placement. Those with two from ${P}$ always have these two leaves together, regardless of the placement of $P$. Since $P$ is rooted, those with three will have the topology implied by the triplet from $P$ regardless of its placement. $\square$

Based on this simple observation, we define:

#### **Definition 2**

For any instance of the Q-SPR problem, a quartet is called *solo* if it includes exactly one leaf from ${P}$. For a solo quartet, removing the sole leaf from $P$ creates its *associated* triplet. A solo quartet is said to belong to an HDT component *X* when the L-type component corresponding to *each* of its four leaves is nested within *X*.

#### Observation 2

Q-SPR problem can be solved by repeated applications of tripVote.

#### Proof

Since only solo quartets matter, we can place each single-leaf tree *q* of ${P}$ independently using tripVote to obtain the quartet score $S_4({B} \overset{v}{\circ }q, R)$ for every node $v \in V_B$. Then, we can compute

$$\begin{aligned} S_4({B} \overset{v}{\circ }P, R) = C + \sum _{t_i \in L_{P}} {S_4( {B} \overset{v}{\circ }t_i, R)} \end{aligned}$$

where *C* is a constant (shared quartets with zero or more than one leaf from $P$), which is independent of the node *v* and thus can be ignored. It is easy to see that by Observation 1, maximizing this score returns the optimal placement. $\square$

Using tripVote to solve the Q-SPR problem requires $O(kd^2(n-m)\log (n-m)\log (n)m)$ time, which is $O(kd^2n^2\log ^2(n))$ for $m=\Theta (n)$ and is thus impractical for large enough *n*. Instead, we propose a new method to directly solve Q-SPR, as summarized in Algorithm 1. This algorithm borrows ideas from tripVote but moves it from being based on triplets to quartets; that is, we directly count the number of shared quartets between the query tree and an arbitrary rooting of the reference tree. Thus, we have to mark the leaves of $P$ on reference tree(s). We assign a new color $-1$ to the leaves of the query subtree $P$ (Fig. 1C). These leaves, which are missing from $B$, have a fixed color. We will also need new counters. Below, we focus on the case with one reference tree *R* but handling multiple reference trees follows trivially.

**Algorithm 1:**
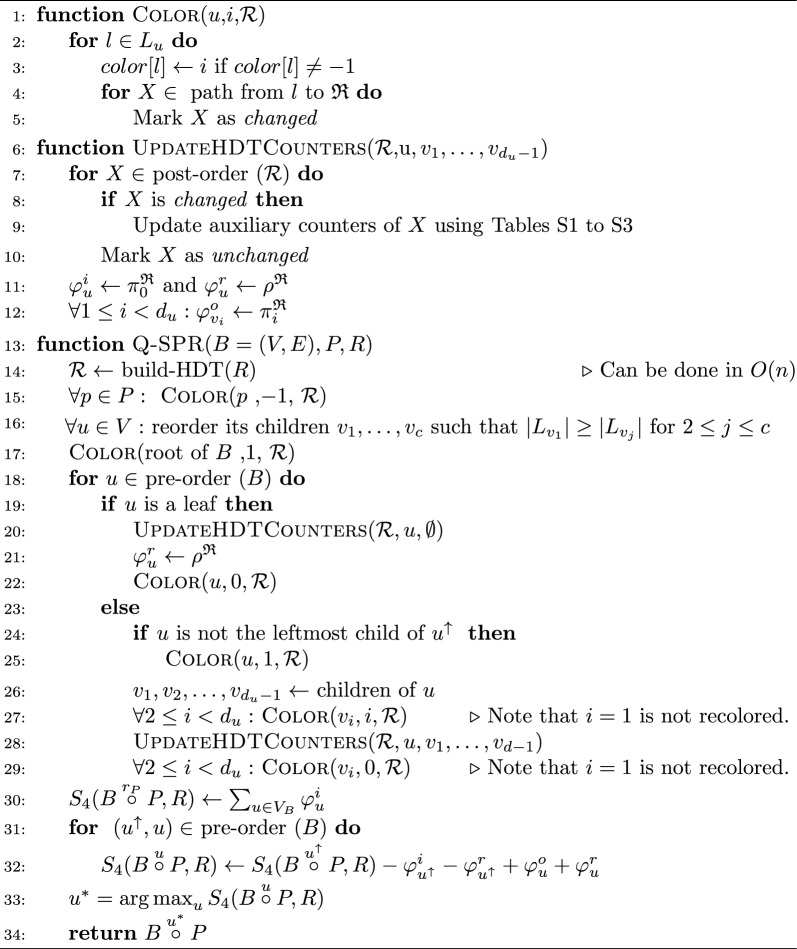
Solution to the Q-SPR problem. $d_u$ is the degree of  *u*.

Algorithm 1 operates through a pre-order traversal of $B$. Visiting each node *u*, it colors leaves of the HDT representing *R* such that each side of *u* has a different color, with 1 assigned to the largest child and 0 assigned to leaves not under *u* (Fig. 1C). Once leaves have the right color, it updates a set of auxiliary counters available on the HDT for all nodes that have been recolored (UpdateHDTCounters function). When these updates are all done, it has computed (line: 11) a set of counters for *u*, as defined in Lemma 1 and depicted in Fig. 1B. Once the traversal of $B$ is finished, using these counters, and the recursion given in Lemma 1 (see Fig. 1B), the algorithm is able to count each quartet shared between *R* and ${B} \overset{u}{\circ }P$. In the end, finding the edge with the maximum score is trivial as the score for each edge is computed.

#### **Lemma 1**

*For any non-root node *$u\in V_{B} {\setminus } \{r_{B}\}$*, the quartet score can be calculated recursively using*

2 $$\begin{aligned} S_4({B} \overset{u}{\circ }P, R) = S_4({B} \overset{u^{\uparrow }}{\circ }P, R) - \varphi _{u^{\uparrow }}^i - \varphi _{u^{\uparrow }}^r + \varphi _u^o + \varphi _u^r \end{aligned}$$

*where* $\varphi _u^i$, $\varphi _u^r$, *and* $\varphi _u^o$ *are defined as the number of shared solo quartets between the placement *${B} \overset{u}{\circ }P$ *and R such that the associated triplet would be anchored at*

- $\varphi _u^i$*: node u in the given rooting of *$B$,
- $\varphi _u^r$*: the new root of the alternative rerooting *$B^\oplus _{u}$,
- $\varphi _u^o$*: node *$u^{\uparrow }$ *in the alternative rerooting of *$B^\oplus _{u}$.

*The quartet score for the root of B*, $r_{B}$, is:

3 $$\begin{aligned} S_4({B} \overset{r_{B}}{\circ }P, R) = C+ \sum _{u \in V_B} {\varphi _{u}^i} \end{aligned}$$

*where C is a constant that can be ignored*.

#### Proof

Recall that according to Observation 1, only the solo quartets play a role in calculating the quartet score when comparing different placements of the query subtree $P$ on the backbone tree $B$. We let the constant *C* include all non-solo quartets that are shared. Thus, we only count shared solo quartets in the other terms. At the root of the backbone tree $B$, it is evident that each shared solo quartet is precisely counted once at the anchor of its associated triplet. Thus, Eq. 3 corresponds to the quartet score resulting from placing $P$ on $r_{B}$.

For any non-root node $u \in V_{B} {\setminus } \{r_{B}\}$, we partition the leaves of $B$ into three sets (those under it, under its sister, and the rest): $L_{{B}^\vee _{u}}$, ${L_{{B}^\vee _{u^{\uparrow }}} {\setminus } L_{{B}^\vee _{u}}}$, and $L_{{B}^\wedge _{u^{\uparrow }}}$. We examine all possible scenarios in which the leaves of the associated triplets for the shared quartets between ${B} \overset{u}{\circ }P$ and *R* can be distributed among these three sets. For each case, Table 3 establishes that the shared quartets are counted precisely once in Eq. 2 (see Fig. 1B). A quartet that is a shared quartet for both ${B} \overset{u}{\circ }P$ and ${B} \overset{u^{\uparrow }}{\circ }P$ (all but last case) is counted by $S_4({B} \overset{u^{\uparrow }}{\circ }P, R)$. In cases given in the second, third, and fourth lines, a quartet is also counted once with a positive sign and once with a negative sign by other counters. In the last case, only $\varphi _u^r$ counts the quartet.$\square$

**Table 3:** Cases for an associated triplet $L_{t} = \{t_1, t_2, t_3\}$ of a shared solo quartet in recursive equation (2). In each case, the quartet is counted exactly once, as shown in column $\sum$

Case	$S_4({B} \overset{u^{\uparrow }}{\circ }P, R)$	$- \varphi _{u^{\uparrow }}^i$	$- \varphi _{u^{\uparrow }}^r$	$\varphi _u^o$	$\varphi _u^r$	$\sum$
$L_{t} \subset L_{{B}^\vee _{u}}$ or $L_{t} \subset {L_{{B}^\vee _{u^{\uparrow }}} \setminus L_{{B}^\vee _{u}}}$ or $L_t \subset L_{{B}^\wedge _{u^{\uparrow }}}$	1	0	0	0	0	1
$L_t \not \subset L_{{B}^\vee _{u}}$, $L_t \not \subset {L_{{B}^\vee _{u^{\uparrow }}} \setminus L_{{B}^\vee _{u}}}$, and $L_t \subset L_{{B}^\vee _{u^{\uparrow }}}$	1	$-1$	0	0	1	1
$L_t \not \subset L_{{B}^\vee _{u}}$, $L_t \not \subset L_{{B}^\wedge _{u^{\uparrow }}}$, and $L_t \subset L_{{B}^\vee _{u}} \cup L_{{B}^\wedge _{u^{\uparrow }}}$	1	0	$-1$	0	1	1
$L_t \not \subset {L_{{B}^\vee _{u^{\uparrow }}} \setminus L_{{B}^\vee _{u}}}$, $L_t \not \subset L_{{B}^\wedge _{u^{\uparrow }}}$, and $L_t \subset L_{{B}^\wedge _{u}}$	1	0	$-1$	1	1	1
$t_1 \in L_{{B}^\vee _{u}}$, $t_2 \in {L_{{B}^\vee _{u^{\uparrow }}} \setminus L_{{B}^\vee _{u}}}$, and $t_3 \in L_{{B}^\wedge _{u^{\uparrow }}}$	0	0	0	0	1	1

We update the meaning of HDT counters $\rho ^X$ and $\pi ^X_j$ compared to tripVote. Assume in the preorder traversal of $B$, we are on node *u*. Then, $\rho ^X$ and $\pi ^X_j$ count the number of solo quartets that belong to the component *X* of the HDT and match the solo quartets in the placement ${B} \overset{u}{\circ }P$ where the associated triplet is anchored at

- $\rho ^X$: the root in the alternative rerooting $B^\oplus _{u}$,
- $\pi ^X_0$: the node *u* in the alternative rerooting $B^\oplus _{u^{\uparrow }}$,
- $\pi ^X_j (j\ge 1)$: the node *u* in the alternative rerooting $B^\oplus _{v_j}$ where $v_j$ is a child of *u*.

Note that rerootings of $B$ are hypothetical (no actual rerooting is performed). Each unrooted quartet in $B$ will have some arbitrary rooting in *R*. Once again, it is easy to see that at the HDT root $\mathfrak {R}$, we can update the counters of the $B$ from the HDT counters:

4 $$\begin{aligned} \varphi _u^r = \rho ^\mathfrak {R} ~~~~~~~~~~~~~ \varphi _u^i = \pi ^\mathfrak {R}_0 ~~~~~~~~~~~~~ \varphi _{v_j}^o = \pi ^\mathfrak {R}_j . \end{aligned}$$

We are now ready to state a key result:

#### **Lemma 2**

*The* $\rho ^X$ *and* $\pi ^X_j$ *counters of the HDT component X* can be updated in $O(d^2)$ *time assuming the counters for children of X are already calculated.*

#### Proof

We derive a set of recursions for each component, which depend on their type (Table 2). Since *L* types include a single leaf and *I* include no leaf, no quartet can belong to them; thus, $\rho ^X$ and $\pi ^X_j$ are set to zero for these components.

Consider a component X of types $IG \rightarrow C$, $CC \rightarrow C$, or $GG \rightarrow G$, each of which has two child components, denoted by $X_1$ and $X_2$. A quartet *q* belongs to *X* of these types if *q* belongs to *X*1, *q* belongs to *X*2, or *q* has at least one leaf from each child of *X*. Thus, if we let $\rho _{comb}^X$ and $\pi _{comb_j}^{X}$ count the part of $\rho ^X$ and $\pi ^X_j$ coming from those quartets with at least one leaf from each child, then, clearly:

$$\begin{aligned} \rho ^X= & {} \rho ^{X_1} + \rho ^{X_2} + \rho _{comb}^X\\ \pi ^X_j= & {} \pi ^{X_1}_j + \pi ^{X_2}_j + \pi _{comb_j}^{X} \end{aligned}$$

For $IG \rightarrow C$ components, $\rho _{comb}^X$ and $\pi _{comb_j}^{X}$ are both 0 because the *I* component is a single internal node and cannot contain any leaves. For other types, we use auxiliary counters, which lead to 62 distinct cases. In short,

- $\rho _{comb}^X$: For these, the rooted quartets must have either ${ ((i,j),(-1,0))}$ or ${ ((0,0),(-1,i))}$ unrooted topologies, where $i, j \in [1, d]$. The recursive equations for these cases are given in Additional file 1: Tables S1 and S2, respectively. Additional file 1: Fig. S1A demonstrates an example of how $\rho ^X$ is computed and also how $\varphi _u^r$ is computed using $\rho ^X$.
- $\pi _{comb_j}^{X}$: For these, the rooted quartets must have the unrooted topology ${ ((i,i),(-1,k))}$ where $i, k \in [0, d]$ and $i, k \ne j$. The recursions for auxiliary counters are given in Additional file 1: Table S3.

Note that we have counted only resolved quartets. And all solo quartets have exactly a single $-1$. Thus, for each HDT component, we have $O(d^2)$ counters each of which is of the form $(0, -1, i, j)$ for $i,j \in [1, d]$. Following all recursions in Additional file 1: Tables S1–S3, we can easily check that the cost of updating all the counters amortized over all counters is constant. This is because most counters require a constant time while a constant number of counters require $O(d^2)$ time. $\square$

We next formally state that the number of leaf colorings is subquadratic.

#### **Lemma 3**

*The total traversal of a tree *$B$ *with N nodes using Algorithm 1 requires a total of *$N \log (N)$ *leaf coloring steps.*

#### Proof

The proof follows from B13 results and in particular its smaller-half trick. Algorithm 1 first colors all nodes as 1, using *O*(*N*) operations. Then, during the traversal, it recolors each node *u* of $B$ only if *u* has a sibling that is larger or the same size; these nodes are colored as 1 when *u* is visited (line 25), as $2\le i \le d$ when $u^{\uparrow }$ is visited (line 27), and as 0 right before existing $u^{\uparrow }$ (line 29). For a tree with *N* leaves, the sum of the number of leaves under all nodes that have a larger or equal-sized sibling is $O\left( N \log (N)\right)$; consider the worst case, a fully balanced tree, where the number is the solution to $f(N) = 2 f({N}/{2}) + {N}/{2}$, which is $N(1+{1}/{2}\log _2(N))$. Thus, the worst-case scenario for the total number of recolored nodes is $O(N \log (N))$. $\square$

#### **Theorem 1**

*Algorithm 1 optimally solves the Q-SPR problem in *$O(k d^2 (n-m) \log (n-m) \log (n))$ *where* *n is the size of the full tree and m is the size of the pruned subtree.*

#### Proof

The HDT data structure has a height of $O(\log (n))$ by design [22]. According to Lemma 2, updating each node of HDT takes $O(d^2)$, and recoloring one node requires traversing the HDT tree from the leaf to the root. Thus, the total time for recoloring a single leaf is $O(d^2 \log (n))$. Moreover, Lemma 3 established that at most $O((n-m) \log (n-m))$ leaf recoloring steps are needed for our backbone tree $B$ of size $n-m$ after pruning. Thus, we need $O(d^2 (n-m) \log (n-m) \log (n))$ operations for leaf recoloring for one reference tree and $O(k d^2 (n-m) \log (n-m) \log (n))$ if we have *k* such trees. By Eq. 4, after recolroing the leaves under a node *u* of $B$ in the HDT, $\rho ^\mathfrak {R}$ and $\pi ^\mathfrak {R}_j$ give us $\varphi _u^i$, $\varphi _u^r$, and $\varphi _u^o$. By Lemma 1, we obtain the score for placing $P$ on each branch using $\varphi _u^i$, $\varphi _u^r$, and $\varphi _u^o$ values and a simple tree traversal; finally, we simply choose the placement branch $(u^{\uparrow },u)$ that maximizes the score. $\square$

### Tree search using quartet SPR moves

Having access to an optimal and efficient SPR move, we can now design a standard hill-climbing tree search to find the quartet median tree with respect to a set of reference trees *R*. The algorithm begins with a starting tree *T*, obtained using any method. We draw non-root nodes of *T* randomly (without replacement) from *some* distribution resulting in a random permutation of non-root nodes of *T*, denoted as $O_T$. For each node $p$ in the order $O_T$, we prune ${T}^\vee _{p}$ from *T* to obtain ${T}^\wedge _{p}$. We solve the Q-SPR problem to determine the optimal placement node $p^*$ of ${T}^\vee _{p}$. If $p^* \ne p$, we place ${T}^\vee _{p}$ on $p^*$ to obtain the improved tree $T^*={{T}^\wedge _{p}} \overset{p^*}{\circ }{T}^\vee _{p}$ and use $T^*$ as the starting tree in the next round. If not, we apply the same approach to the complementary subtree of ${T}^\vee _{p}$, namely ${(T^\oplus _{p})}^\vee _{p^{\uparrow }}$. Let $p^\star$ be the optimal placement of ${(T^\oplus _{p})}^\vee _{p^{\uparrow }}$ on ${T}^\vee _{p}$. Similarly, if $p^\star \ne p$, we define the improved tree as $T^\star ={{T}^\vee _{p}} \overset{p^\star }{\circ }{(T^\oplus _{p})}^\vee _{p^{\uparrow }}$, using $T^\star$ as the starting tree in the subsequent round. We repeat this process for every node in $O_T$ until an improvement is obtained. The search stops if, in a round, we perform SPR moves on every node of *T* without finding an improvement in the quartet score. Given that the starting tree *T* has $2n-1$ nodes, and we may need to find the optimal placement for all subtrees and their complementary subtrees, the total running time for one round of SPR for all nodes of *T* has a complexity of $O(kd^2n^2\log ^2(n))$.

To fully specify this algorithm, we need to specify the distribution under which we draw without replacement (i.e., permutate) nodes of the tree. A simple choice is a uniform distribution, and we will explore that choice. However, our experiments in section E3. Design of heuristics reveal intriguing empirical patterns regarding the probability of an SPR move improving the quartet score. To make our search algorithm faster, we explored heuristics that assign higher probabilities to nodes with a higher empirical probability of improving the tree. Each node $u \in V_T$ is assigned a weight $w_u$ calculated using a combination of various heuristics explained below. Each heuristic assigns a value $h(u) \in (0,1]$ to *u*. When multiple heuristics are employed, these values are summed to yield the final weight $w_u$ of a node. Subsequently, these weights are normalized using $\hat{w_u} = w_u/\sum _{v \in V_T}{w_v}$. At the beginning of each round of the search algorithm, to obtain the random permutation $O_T$, determining the order in which nodes of *T* are visited in that round, each node *u* is drawn without replacement with probability $\hat{w_u}$. We use three heuristics, as explained below.

*Size of the subtree and its surrounding subtrees.* The outcomes of our experiments reveal an interesting trend in the early stages of the search: Subtrees with sizes closer to *n*/2 or possessing siblings with this characteristic tend to have a higher probability of enhancing the tree. Nevertheless, as the score of the tree improves, only very small or very large subtrees appear to significantly influence the quartet score. For a non-root node $u \in V_T$ and its siblings $\{s_1,..., s_{d-1} \} \subset V_T$ where *d* is the degree of $u^{\uparrow }$, Let $|L_{{T}^\vee _{u}}|$ and $|L_{{T}^\vee _{s_1}}|,..., |L_{{T}^\vee _{s_{d-1}}}|$ be the size of the subtree corresponding to *u* and its siblings, respectively. We define the subtree impact $i_u$ as:

$$\begin{aligned} i_u = \max _{v \in \{u, s_1,..., s_{d-1}\}}\left( {\min {\left( |L_{{T}^\vee _{v}}|, n - |L_{{T}^\vee _{v}}|\right) } } \right) \end{aligned}$$

The heuristic $h_1(u)$ is defined using the subtree impact $i_u$. In practice, we noticed that prioritizing subtrees with larger $i_u$ score in the initial round and subtrees with smaller subtree impact in the later rounds (e.g., $round > 10$) results in the best running time. Thus, we assign $h_1(u)$ using a sigmoid function as follows:

5 $$\begin{aligned} h_1(u) = {\left\{ \begin{array}{ll} \left( 1 + \exp \left( {-10 (\frac{i_u - 1}{n/2} - 0.5)}\right) \right) ^{-1} &{} {(round = 1)} \\ 1 &{} round \in [2,10] \\ 1 - \left( 1 + \exp \left( {-10 (\frac{i_u - 1}{n/2} - 0.5)}\right) \right) ^{-1} &{} {(round \ge 11)} \end{array}\right. } \end{aligned}$$

*Distance from the source and destination of the previous round.* Our findings in section E3. Design of heuristics also indicate that the subtrees around the two nodes associated with the applied SPR move in a given round have a higher likelihood of enhancing the tree in the subsequent round. Let ${T}^\vee _{p}$ be the subtree that moved in the previous SPR round. Let *ps* and $pd \in V_T$ correspond to the parent of the root of ${T}^\vee _{p}$ before and after the SPR move, respectively. For a node $u \in V_T$, we define $D_T(u, ps)$ as the number of edges on the undirected path from the sister of *u* to *ps* in *T*. Similarly, $D_T(u, pd)$ is defined as the nodal distance between the sister of *u* and *pd*. The reason we use distance to the sister is that after a node *u* is pruned, *u* and its parent $u^{\uparrow }$ will be absent, so, the sister to *u* is the closest remaining node. Similarly, *ps* and *pd* correspond to the nodes of *T* that were unchanged when performing SPR move on ${T}^\vee _{p}$ and are the sister to $p$ before and after being moved, respectively. Therefore, $D_T(p, pd) = 0$.

We define two heuristics $h_2(u)$ and $h_3(u)$ based on $D_T(u, ps)$ and $D_T(u, pd)$, respectively, as:

Distance from ps $$\begin{aligned} h_2(u) = 1 - \left( {1 + \exp \left( -10 (\frac{D_T(u, ps)}{2\log {n}} - 0.5)\right) }\right) ^{-1} \end{aligned}$$

Distance from pd $$\begin{aligned} h_3(u) = 1 - \left( {1 + \exp \left( -10 (\frac{D_T(u, pd)}{2\log {n}} - 0.5)\right) }\right) ^{-1} \end{aligned}$$

For the initial round ($round = 1$), we set $h_2(u) = 1$ and $h_3(u) = 1$ for all $u \in V_T$. In a specific scenario where $D_T(u, pd) = 0$, signifying that either ${T}^\vee _{u}$ or ${T}^\wedge _{u}$ is the subtree on which the last round’s SPR move was applied, leading to a minimal chance of improvement for the node *u*, we assign a very small weight to *u* by setting $D_T(u, pd) = 2\log {n}$.

**Fig. 2:**
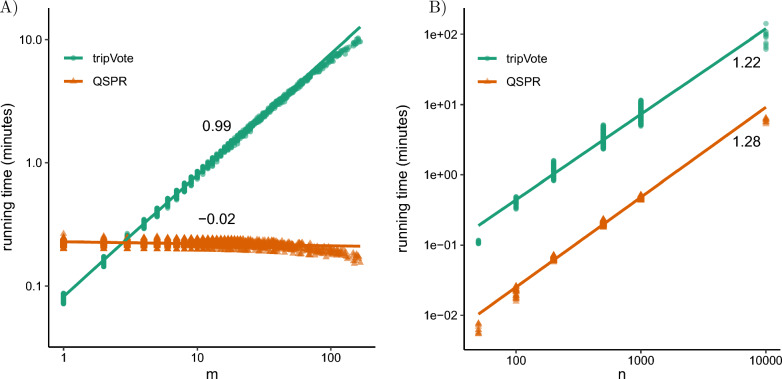
E1 Results. Running time versus **A** the query subtree size (*m*) or **B** the total tree size (*n*). We fix $n=500$ in **A** and limit $30< m < 70$ in **B**. Figures are log-log scale. Thus, the shown line slope is an empirical estimate of the asymptotic running time polynomial degree. Here, tripVote refers to the method described in Observation 2

*Starting tree*. The user can input a starting tree, or, in cases where such a tree is not provided, we employ the following strategy to construct one: Beginning with a rooted three-taxon tree, where the taxa are randomly selected from the leaf set of the reference trees, we iteratively employ tripVote to place each additional taxon with respect to the reference trees until a complete tree is formed. We opt for tripVote in this process because, as evidenced by our experiments (refer to Fig. 2), it demonstrates faster performance when the query subtree is particularly small ($|L_P| \le 2$). We use tripVote in the default setting which also subsamples short quartets.

*Implementation details*. Our implementation of the Q-SPR algorithm, along with a comprehensive search-based method for finding the median quartet tree given a set of reference trees is publicly accessible on GitHub. This code is built upon tripVote, which, in turn, relies on the tqDist library [32]. tqDist is a library that calculates triplet and quartet scores between two trees, employing the B13 algorithm.

### Experimental setup

We include four experiments. E1) An analysis of the time complexity of the Q-SPR algorithm, comparing it to a modified version of tripVote capable of quartet-based SPR moves. E2) A comparison of the hill-climbing search method for finding the median quartet tree with the state-of-the-art tool ASTRAL-III. E3) Exploration of various heuristic approaches to enhance Q-SPR speed. E4) The use of Q-SPR as a subsequent step to widely-used tree estimation tools like ASTRAL-III and ASTER to further enhance their optimality and accuracy.

#### E1: Running time comparison

We compared Q-SPR to a modified version of tripVote, as described in Observation 2. We used an existing 10,000-taxon simulated dataset [33] including 10 replicates with gene trees disagreeing with the species tree due to both ILS and Horizontal Gene Transfer (HGT), as simulated by SimPhy [34]. We used the true species tree as the query tree *T* and the available gene trees estimated using FastTree-II [35] as the reference set. Note that the gene trees include polytomies. We randomly selected $n\in \{ 50, 100, 200, 500, 1000, {10000}\}$ taxa for each replicate and pruned both query trees and the reference trees to contain only the selected *n* taxa. We also subsampled the gene trees randomly to obtain $k = 100$ trees per replicate. For each replicate, we applied a single round of SPR on every subtree $P$ of the query tree *T* and measured the time each method took to find the optimal placement. For trees of size $n \ge 1000$, we restricted these analyses to subtrees of size $m \le 70$. In addition to the running times, we computed the quartet score $S_4({{T}^\wedge _{p}} \overset{v}{\circ }P, R)$ for every node *v* of ${T}^\wedge _{p}$ and compared the scores of Q-SPR to the scores of modified tripVote to ensure the correct implementation of Q-SPR.

#### E2. Full quartet median tree search using heuristics

In this experiment, we tested the performance and accuracy of our hill-climbing search method in comparison to ASTRAL-III. The dataset used for this experiment was an existing Simphy-simulated ILS-only 200-taxon dataset [21], simulated under three levels of ILS (tree height $\in \{5\times 10^5, 2\times 10^6, 10^7\}$ corresponding to high, medium, and low levels of ILS, respectively). For each ILS level, we considered the 50 replicates with the speciation rate of $10^{-6}$ and a set of either 50 or 200 estimated gene trees as our reference tree set. These gene trees can have polytomies, and trees that have less than twice the number of nodes of a fully resolved tree are removed, as done by Mirarab and Warnow [21]; thus, the actual input can include fewer than 50 or 200 gene trees. We generated the starting trees from the reference set using the method described in section Tree search using quartet SPR moves. For each replicate, we evaluated our optimization method compared to ASTRAL-III in terms of the normalized quartet score of the final tree with respect to the gene trees (i.e., the optimization score). We also compared the accuracy of the tree produced by the two methods, comparing them to the true species tree in terms of quartet score [3] and RF (Robinson-Foulds) distance [36]. Finally, we compared the running time of our method, including the building of the starting tree, with the running time of ASTRAL-III.

#### E3. Exploring heuristic approaches

To develop heuristics for improving the effectiveness of moving each subtree, we investigated how characteristics of each subtree predict the likelihood of enhancing the quartet score when moved to the optimal position. We changed the search algorithm to execute all feasible SPR moves in each round, recording the improvement in the quartet score if any was achieved without updating the query tree. At the conclusion of each round, the SPR move with the greatest improvement was applied to the query tree. For each subtree, we examined characteristics such as the size of the subtree and its neighboring subtrees, the nodal distance from the root of the subtree to the nodes associated with the optimal SPR move in the preceding round, and the number of applied SPR moves (i.e., the number of completed rounds). These were chosen among a larger set of metrics examined (not shown) which did not show as much predictive power. Subsequently, we explored whether these characteristics can predict the likelihood of improvements in the score resulting from an SPR move. Based on the outcomes of this experiment (section E3. Design of heuristics), we formulated three heuristic functions outlined in section Tree search using quartet SPR moves. We investigated how any combination of these functions affects the running time of our search algorithm. Where not explicitly specified, the combination of all three methods was utilized as the default method. Additionally, we explored whether the use of heuristics accelerates the convergence of the search algorithm to the optimal score.

#### E4. Improving ASTER and ASTRAL-III trees

We conducted an experiment using the highly optimized trees produced by ASTER and ASTRAL-III as inputs for our search algorithm to assess whether the optimality and accuracy of these trees could be further improved through additional SPR moves. In this experiment, we evaluated ASTRAL-III and ASTER, with the latter being a newer algorithm shown to outperform ASTRAL-III in handling missing data [24]. We used the gene trees made available by Zhang and Mirarab [24] who modified the 200-taxon dataset of Mirarab and Warnow [21] used in E2 to remove approximately 5% of taxa at random from each estimated gene tree. We used this version with missing data because Zhang and Mirarab [24] documented that the presence of only a small number of missing data can impact the optimality of ASTRAL-III. Other settings of the dataset are identical to E2.

To measure improvements after running Q-SPR, we compared the starting tree and its output against the gene trees and the true known species tree. We report the quartet score and the normalized Robinson-Foulds (nRF) [36] metric. In addition, we used ASTRAL-III to compute the local posterior probability (PP) [37] and the coalescent unit length for each internal branch for each tree with respect to the gene tree. ASTRAL-III sets branch lengths to zero when the frequency of the species tree quartet topology is less than ${1}/{3}$ among gene tees, a pattern that is unexpected under MSC with a large enough number of gene trees. Similarly, localPP would be set to less than ${1}/{3}$ under those conditions. We evaluated support and length for internal branches, with a particular focus on the unexpected cases with zero branch length or localPP less than ${1}/{3}$.

## Results

### E1: running time versus *m* and *n*

Theoretical asymptotic results match our empirical running time measurements (Fig. 2). Recall that the running time of Q-SPR for moving a subtree of size *m* from a tree of size *n* is $O(k (n-m) \log (n-m) \log (n))$ compared to $O(k m (n-m) \log (n-m) \log (n))$ for tripVote. For fixed $n=500$ and changing *m*, the asymptotic advantage of Q-SPR over tripVote is clear (Fig. 2A). Matching theory, the running time of Q-SPR is nearly independent of *m* (ranging between 9 and 16 seconds with a mean of 13.5). The running time of tripVote increases linearly with *m*, again, as expected. Interestingly, tripVote is faster for subtrees of size one or two ($m \le 2$). This is because tripVote has fewer counters to maintain than Q-SPR, and thus has a smaller constant factor. The benefit of Q-SPR appears only for larger *m* values; e.g., for $m>100$, Q-SPR is 73 times faster than tripVote on average. Note that the Q-SPR running time reduces slightly with *m*. To understand why, note that increasing *m* decreases the size of the backbone tree $B$, which is $n-m$. Thus, as *m* increases, Q-SPR can become faster because it depends on the size of $B$ and not *m*.

**Table 4:** E2 Results.

Genes	ILS-Level	Normalized quartet score		Normalized RF	
		ASTRAL-III	Q-SPR	ASTRAL-III	Q-SPR
$k=50$	High	$0.8810 \pm 0.087$	$0.8853 \pm 0.089$	$0.1767 \pm 0.048$	$0.1818 \pm 0.054$
	Med	$0.9572 \pm 0.053$	$0.9575 \pm 0.053$	$0.0845 \pm 0.041$	$0.0843 \pm 0.043$
	Low	$0.9871 \pm 0.026$	$0.9871 \pm 0.026$	$0.0515 \pm 0.036$	$0.0519 \pm 0.037$
$k=200$	High	$0.9617 \pm 0.041$	$0.9589 \pm 0.042$	$0.0942 \pm 0.037$	$0.0953 \pm 0.039$
	Med	$0.9720 \pm 0.052$	$0.9740 \pm 0.047$	$0.0501 \pm 0.043$	$0.0496 \pm 0.043$
	Low	$0.9928 \pm 0.019$	$0.9928 \pm 0.019$	$0.0308 \pm 0.029$	$0.0309 \pm 0.030$

Comparing the accuracy of Q-SPR and ASTRAL-III in terms of normalized quartet score and RF distance to the true species tree

When we change the tree size *n* and apply SPR to mid-size subclades ($30< m < 70$), tripVote and Q-SPR have similar running time growth rates (Fig. 2B). This is because the theoretical running time of both methods is quasi-linear with respect to *n*; empirically, the observed log-log slope is slightly above 1.0, matching these expectations. However, note that for all *n*, Q-SPR is faster than tripVote by 10 to 31 times (mean: 16). This pattern also matches the theoretical expectations because Q-SPR is faster than tripVote by a factor of $\Theta (m)$.

### E2: Tree estimation using Q-SPR search

**Fig. 3:**
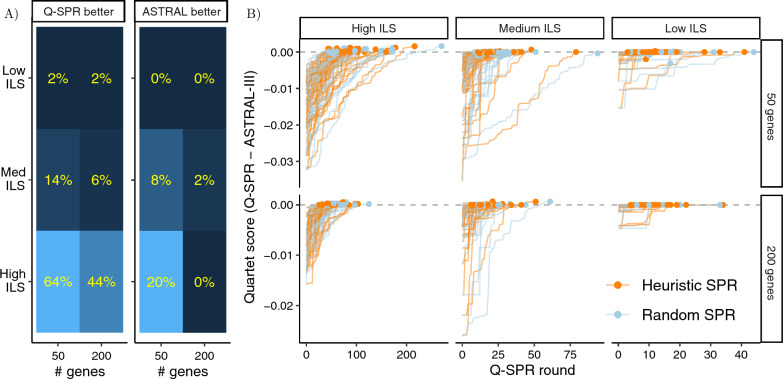
E2 Results. **A** Percentage of the replicates with improved (left) and reduced (right) quartet scores compared to ASTRAL-III. These results are based on the heuristic search. **B** We show the normalized quartet score of Q-SPR trees across rounds minus the normalized quartet score of the ASTRAL-III tree (both with respect to input gene trees) as rounds progress (0: starting tree). The final delta normalized quartet score compared to the ASTRAL is marked for each replicate.

Q-SPR obtains a better optimization score than ASTRAL-III in 66 out of 294 cases, while ASTRAL-III has a better score in 15 cases (Fig. 3A). Moreover, Q-SPR improvements are more substantial than ASTRAL. Averaged over all cases, Q-SPR achieves a 0.012% higher normalized quartet score than ASTRAL, with this difference being close to 0.061% when considering only the cases where Q-SPR outperforms ASTRAL. These improvements are particularly significant for the high ILS (tree height $= 5 \times 10^5$) and $k=50$ model condition, showing a 0.068% improvement overall and 0.092% for the cases with a higher score. These improvements in quartet scores are despite the fact that the starting trees of Q-SPR have substantially lower scores—often 1–3% (Fig. 3B). In all but a handful of cases, Q-SPR manages to reach scores close to or above ASTRAL-III. The number of rounds of SPR needed ranges from as few as 4 and as high as 271, with more rounds needed for higher ILS and fewer genes.

Enhancing the quartet score with respect to the gene trees does not meaningfully impact the tree accuracy (Table 4). Exact conditions where one method outperforms the other depend on the choice of the metric, but in all cases, changes in accuracy are small compared to variation across replicates. Only 35% and 59% of the cases with improved quartet scores also exhibit higher accuracy compared to ASTRAL-III in terms of nRF and quartet score.

In terms of running time, ASTRAL-III is substantially faster than the Q-SPR search (Additional file 1: Table S4). On average, ASTRAL is 124 and 306 times faster than Q-SPR search for $k=50$ and $k=200$, respectively. It is important to note that although ASTRAL-III is faster in practice, all our inputs had low number of genes *k*. The running time of one round of Q-SPR grows linearly with the number of genes *k*, while ASTRAL-III running time grows quadratically with *k*.

### E3. Design of heuristics

**Fig. 4:**
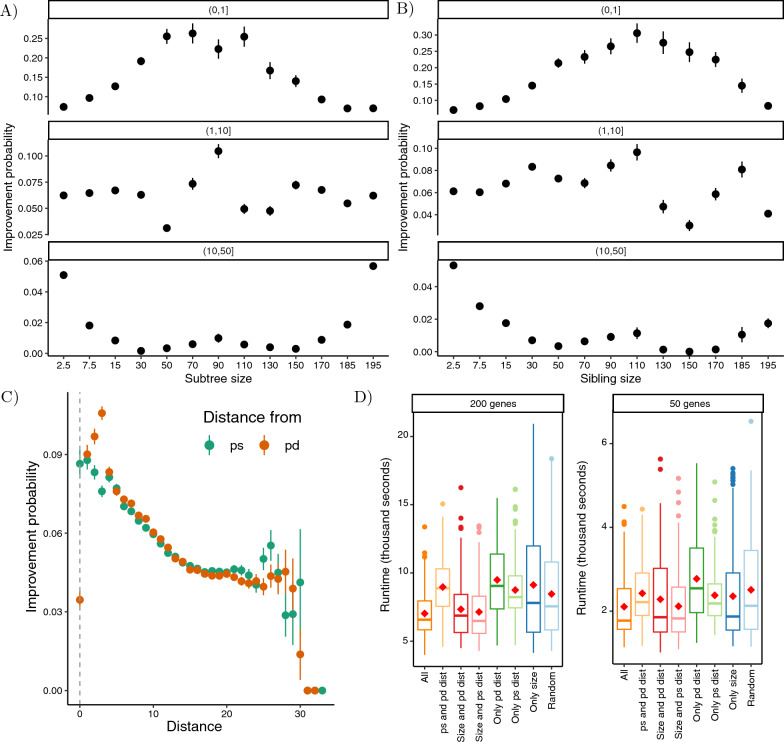
E3 Results. **A**, **B** The impact of the size of a subtree (**A**) or its sister (**B**) on the probability that an SPR applied to that subtree leads to an improved quartet score. Panels show the impact on the first round, middle rounds (1, 10], and final runs (10, 50]. The starting tree is the result of step-wise additions. In the first round, subtrees with a size around ${n}/{2}$ have a higher probability of improvement while in the final rounds, small and larger subtrees are likely to improve speed. **C** Improvement probability of an SPR move compared to the distance to the source (*ps*) or the destination (*pd*) of the previous move. For each node *p*, we show the distance from its sister (i.e., the closest node left in the tree after we remove ${T}^\vee _{p}$) to the node above which the previous SPR move was placed (*pd*) or the sister of the node that was moved in the previous SPR move (*ps*). Subtrees close to the previous source or destination have a higher probability of improving the score. The reduction at distance 0 for *pd* is because this case represents an attempt to move the previously moved node *p*, or its complement ${T}^\wedge _{p}$, and the former by construction has 0 probability of moving because it is already in its optimal position. **D** Comparison of the running time for a full Q-SPR search run between different combinations of heuristic methods. The building time for the starting tree is also included.

The size of the pruned subtree has predictive power for the probability of improvement of an SPR move in ways that change with the number of passed rounds (Fig. 4A). In the initial rounds, the subtrees with a size closer to *n*/2 have a higher likelihood of improving the tree when moved by an SPR move. However, as the rounds progress and the tree becomes closer to optimal, this pattern reverses, and subtrees with either very small or very large sizes become more likely to improve the score. This observation can be explained. An SPR move on mid-sized subtrees contributes the most to change in tree topology and change in the quartet score. In the initial rounds, when the tree is far from optimal, moving these subtrees exerts the most impact. However, as the search progresses and the tree becomes closer to optimal, these mid-sized subtrees become less likely to move. Consequently, very small or very large subtrees gain higher chances of improvement in later rounds. Note that very small and very large subtrees are equivalent because at each node *p*, we examine moving both ${T}^\vee _{p}$ and ${T}^\wedge _{p}$. The same pattern is observed when considering the impact of the size of the sibling of the pruned node on the likelihood of improvement (Fig. 4B). These observations are the impetus for the first heuristic given in (5).

Another intriguing pattern is the impact of the distance of a subtree from the areas affected by the SPR move in the previous round (Fig. 4C). Our experiment suggests a clear correlation between these distances and the likelihood of improvement. Nodes close to either the source or the destination of the previous successful SPR move have a higher chance of having a successful SPR of their own. This result is the basis for heuristics $h_2$ and $h_3$.

The set of three heuristics results in moderate reductions in running time (Fig. 4D). Using all of the three heuristics described in section Tree search using quartet SPR moves reduces the running time by 15 min on average (Additional 1: Table S4). Interestingly, it appears that combining at least two of the three metrics is needed to obtain improved speeds. Our results from E2 also show that using heuristic approaches results in a faster optimization of the tree in terms of the number of rounds (Fig. 3B).

### E4. Improving ASTER and ASTRAL-III trees

**Fig. 5:**
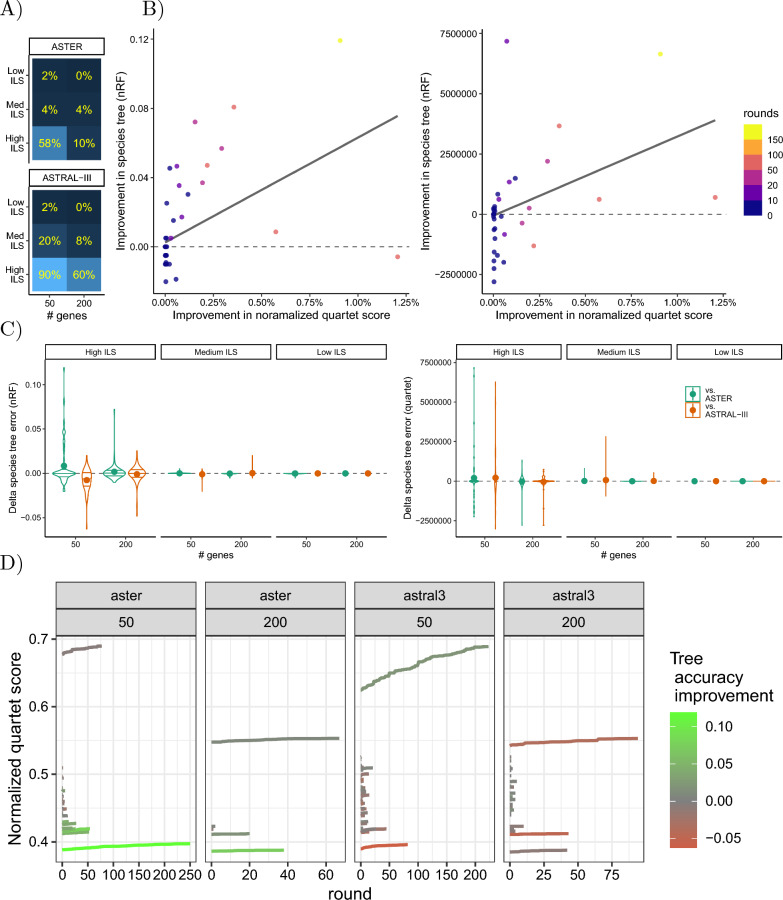
E4 Results. **A** For each input model condition, we show the percentage of the replicates with improved quartet scores, starting from either ASTER or ASTRAL-III trees. **B** Improvement in the quartet score of the Q-SPR algorithm above the ASTER tree (under the High ILS model condition and $k=50$) with respect to the gene trees versus the improvement in the normalized RF or the quartet distance between the ASTER tree and the true species tree. The number of SPR rounds performed for each replicate is shown in colors. Restricted to high ILS, 50 genes, ASTER; see Additional file 1: Fig. S3 for all model conditions. **C** The difference between the normalized RF and the quartet distance of the Q-SPR tree and the starting tree with respect to the true species tree for all tested conditions. Positive (negative) values indicate an improvement (reduction) in the accuracy of the starting tree. **D** The normalized quartet score between the Q-SPR tree at the end of each SPR round and the gene trees under the High ILS (ILS rate $= 500k$) model conditions. The final improvement of the Q-SPR tree with respect to the true species tree is shown in colors. See Additional file 1: Fig. S2 for full results.

Out of 600 replicate runs tested, Q-SPR improves the quartet score (i.e., the optimization criterion) compared to the ASTRAL-III or ASTER starting trees in 129 replicates. However, patterns of improvement in the quartet score depend heavily on the level of ILS and the starting tree method (Fig. 5A). ASTRAL-III is improved more than ASTER, consistent with results of [24], showing that ASTRAL-III output can be suboptimal for cases with missing data and relatively few input trees. Also, improvements are more pronounced for 50 genes compared to 200 and for medium ILS compared to low ILS. While the improvement in quartet score can be up to 6% in rare cases, in most cases it is under 0.5% (Additional file 1: Fig. S3).

Better optimization scores do not consistently lead to substantially improved species trees (Fig. 5B). For low and medium ILS, accuracy rarely changes, while some improvements are observed for high ILS, in particular with respect to ASTER (Fig. 5C). Out of the 129 cases with improved quartet scores, the Q-SPR tree was more accurate in only 45 or 68 cases, in terms of nRF or quartet distance, respectively. However, in a substantial number of cases (84 or 61, for nRF and quartet distance, resp.), the improved optimization score led to reduced accuracy. It should be noted that when the quartet score improves but accuracy degrades, the reductions are small (mean: 0.96% nRF). When the quartet score does improve, the improvements in accuracy can be up to 11.9% (mean: 1.91% nRF). Cases with high accuracy improvement tend to be those with higher increases in the quartet score (Fig. 5B and Additional file 1: Fig. S3). Beyond accuracy, local support values also change as a result of running Q-SPR (Additional file 1: Fig. S4). In particular, the output trees include fewer branches that have support below ${1}/{3}$ and branch length 0, which is not expected under the MSC model. Interestingly, it appears that compared to ASTER, Q-SPR has more branches with 100% support as well.

The progress of Q-SPR across rounds shows high variation across replicates and model conditions (Additional file 1: Fig. S2). While the mean number of rounds is 1 for low ILS, 200 genes condition, for high ILS, 50 genes, we need 13.2 rounds on average. It also appears that improved accuracy is often obtained in challenging datasets where the quartet score is low, to begin with, while substantial improvements in quartet score often do not improve accuracy if the initial quartet score is high (Fig. 5E).

## Discussion

Our main algorithmic contribution in this paper was showing how to find the optimal SPR move for quartet distance in time that grows quasi-linearly with the size of the tree. The best previous method for solving this method was repeated applications of tripVote (Observation 2), which is asymptotically slower than our method by a factor of *n*. Using our efficient algorithm for SPR moves, we were able to build a hill-climbing method for inferring species trees from gene trees.

Our resulting method, Q-SPR, was slower than ASTRAL-III and no more accurate than it. While this observation reduces the immediate impact of Q-SPR in practice, it does get us close to answering a fundamental question: Is the combined scalability and accuracy of ASTRAL-III due to its dynamic programming algorithm? The answer seems to be yes, as employing the traditional hill-climbing approach achieves essentially the same accuracy but at a much higher running time. The implication of this observation for future work is that perhaps using a dynamic programming algorithm constrained to a predefined search space for phylogenetic inference problems other than quartet median tree can improve their scalability and accuracy as well.

We showed that Q-SPR can improve on ASTRAL-III and ASTER in terms of the objective function if those are used as the starting tree. On a practical level, these improvements are useful as they eliminate cases with very low local posterior probability support, particularly those with support below ${1}/{3}$. However, the fact that topological accuracy does not improve despite improvements in quartet score is interesting. The reason seems to be that branches that change tend to be low support branches that are uncertain in both resolutions. In other words, the imperfect correlation between the quartet score and accuracy given a limited number of genes has reduced the impact of improving the quartet score beyond what heuristics such as ASTER and ASTRAL-III achieve. In practice, the main benefit of following ASTRAL-III or ASTER with Q-SPR is to 1) test whether differences between outputs of these methods and alternative analyses (e.g., concatenation) can be explained by lack of optimality as opposed to other explanations, and 2) eliminate problematic branches with 0 length or support $<{1}/{3}$.

In a hill-climbing search, if one can prioritize what branches to examine, the search may converge faster. We identified some potential ways of making such predictions and observed moderate improvements in running time as a result. We leave it to future work to examine whether more elaborate methods, such as those proposed by Azouri et al. [38] can further improve accuracy. Such future work should also examine the impact of starting from different types of starting trees or multiple starting trees, which may impact accuracy (and will impact running time). Beyond prioritizing SPR moves, interesting theoretical questions remain unanswered: Perhaps some of the calculations performed in one SPR round can be reused in the next round, or calculations for one clade could be reused for adjacent clades. Moreover, we did not attempt NNI moves, but those are a special case of SPR, and perhaps those can be implemented with reduced computational requirements. Finally, our implementation of the search heuristic (as opposed to the Q-SPR move) is based on a Python code without extensive performance optimization. Future work can further optimize the code and explore parallelism.

### Supplementary Information

**Additional file 1. **The supplementary materials include additional tables and figures referenced in this paper.

## Data Availability

The code is publically available at (github.com/shayesteh99/QuartetSPR). The data generated during this study is available in (github.com/shayesteh99/QuartetSPR-Data).
