# Supplementary material for: Median quartet tree search algorithms using optimal subtree prune and regraft
Source: Algorithms Mol Biol. 2024 Mar 13;19:12. doi: 10.1186/s13015-024-00257-3 (PMC10938725; doi:10.1186/s13015-024-00257-3)
Supplement: Supplementary file 1 — Additional file 1. The supplementary materials include additional tables and figures referenced in this paper. [file 13015_2024_257_MOESM1_ESM.pdf]

Supplementary Materials for Median Quartet Tree Search Algorithms using Optimal Subtree Prune and Regraft

Shayesteh Arasti Siavash Mirarab

1 Supplementary Tables

Rooted Quartet		$CC \to C$		$GG \to G$
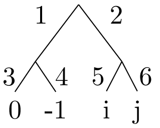	1<br>2<br>3<br>4<br>5, 6	$n_0^{C_1} n_{-1}^{C_1} [n_{(\bullet\square)}^{C_2} + n_{(\bullet\bullet)}^{C_2}]$<br>$+\binom{n_\bullet^{C_1}}{2} n_{(0-1)}^{C_2}$<br>$+n_0^{C_1} [n_{-1\uparrow(\bullet\square)}^{C_2} + n_{-1\uparrow(\bullet\bullet)}^{C_2}]$<br>$+n_{-1}^{C_1} [n_{0\uparrow(\bullet\square)}^{C_2} + n_{0\uparrow(\bullet\bullet)}^{C_2}]$<br>$+n_\bullet^{C_1} n_{\bullet\uparrow(0-1)}^{C_2}$	1<br>2	$n_{(0-1)}^{G_1} [n_{(\bullet\square)}^{G_2} + n_{(\bullet\bullet)}^{G_2}]$<br>$+n_{(0-1)}^{G_2} [n_{(\bullet\square)}^{G_1} + n_{(\bullet\bullet)}^{G_1}]$
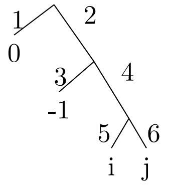	1<br>2<br>3<br>4<br>5, 6	$+n_0^{C_1} [n_{(-1(\bullet\square))}^{C_2} + n_{(-1(\bullet\bullet))}^{C_2}]$<br>$+[n_{[-1(\bullet\square)]}^{C_1} + n_{[-1(\bullet\bullet)]}^{C_1}] n_0^{C_2}$<br>$+n_{-1}^{C_1} [n_{(\bullet\square)\uparrow 0}^{C_2} + n_{(\bullet\bullet)\uparrow 0}^{C_2}]$<br>$+\binom{n_\bullet^{C_1}}{2} n_{-1\uparrow 0}^{C_2}$<br>$+n_\bullet^{C_1} n_{\bullet\uparrow -1\uparrow 0}^{C_2}$	1<br>2	$+n_0^{G_1} [n_{[-1(\bullet\square)]}^{G_2} + n_{[-1(\bullet\bullet)]}^{G_2}]$<br>$+n_0^{G_2} [n_{[-1(\bullet\square)]}^{G_1} + n_{[-1(\bullet\bullet)]}^{G_1}]$
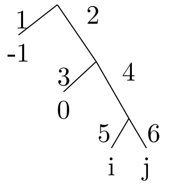	1<br>2<br>3<br>4<br>5, 6	$+n_{-1}^{C_1} [n_{(0(\bullet\square))}^{C_2} + n_{(0(\bullet\bullet))}^{C_2}]$<br>$+[n_{[0(\bullet\square)]}^{C_1} + n_{[0(\bullet\bullet)]}^{C_1}] n_{-1}^{C_2}$<br>$+n_0^{C_1} [n_{(\bullet\square)\uparrow -1}^{C_2} + n_{(\bullet\bullet)\uparrow -1}^{C_2}]$<br>$+\binom{n_\bullet^{C_1}}{2} n_{0\uparrow -1}^{C_2}$<br>$+n_\bullet^{C_1} n_{\bullet\uparrow 0\uparrow -1}^{C_2}$	1<br>2	$+n_{-1}^{G_1} [n_{[0(\bullet\square)]}^{G_2} + n_{[0(\bullet\bullet)]}^{G_2}]$<br>$+n_{-1}^{G_2} [n_{[0(\bullet\square)]}^{G_1} + n_{[0(\bullet\bullet)]}^{G_1}]$
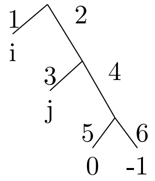	1<br>2<br>3<br>4<br>5<br>6	$+n_\bullet^{C_1} n_{(\bullet(0-1))}^{C_2}$<br>$+n_{[\bullet(0-1)]}^{C_1} n_\bullet^{C_2}$<br>$+n_\bullet^{C_1} n_{(0-1)\uparrow\bullet}^{C_2}$<br>$+n_0^{c_1} n_{-1}^{C_1} [n_{\bullet\uparrow\square}^{C_2} + n_{\bullet\uparrow\bullet}^{C_2}]$<br>$+n_0^{C_1} [n_{-1\uparrow\bullet\uparrow\square}^{C_2} + n_{-1\uparrow\bullet\uparrow\bullet}^{C_2}]$<br>$+n_{-1}^{C_1} [n_{0\uparrow\bullet\uparrow\square}^{C_2} + n_{0\uparrow\bullet\uparrow\bullet}^{C_2}]$	1<br>2	$+n_\bullet^{G_1} n_{[\bullet(0-1)]}^{G_2}$<br>$+n_\bullet^{G_2} n_{[\bullet(0-1)]}^{G_1}$

Table S1: **Equations for computing the HDT counter ρ^X_{comb} for component types $CC \to C$ and $GG \to G$.** Each row shows one rooted topology of the unrooted quartet $((i,j),(-1,0))$. ρ^X_{comb} for each component type is the sum over the equations of all the rooted topologies.

Rooted Quartet		$CC \rightarrow C$		$GG \rightarrow G$
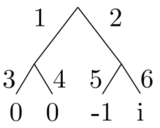	1<br>2<br>3, 4<br>5<br>6	$+\binom{n_0^{C_1}}{2} n^{C_2}_{(-1\bullet)}$<br>$+n^{C_1}_{-1} n^{C_1}_{\bullet} n^{C_2}_{(00)}$<br>$+n^{C_1}_{0} n^{C_2}_{0\uparrow(-1\bullet)}$<br>$+n^{C_1}_{-1} n^{C_2}_{\bullet\uparrow(00)}$<br>$+n^{C_1}_{\bullet} n^{C_2}_{-1\uparrow(00)}$	1<br>2	$+n^{G_1}_{(00)} n^{G_2}_{(-1\bullet)}$<br>$+n^{G_2}_{(00)} n^{G_1}_{(-1\bullet)}$
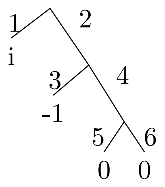	1<br>2<br>3<br>4<br>5, 6	$+n^{C_1}_{\bullet} n^{C_2}_{(-1(00))}$<br>$+n^{C_1}_{[-1(00)]} n^{C_2}_{\bullet}$<br>$+n^{C_1}_{-1} n^{C_2}_{(00)\uparrow\bullet}$<br>$+\binom{n_0^{C_1}}{2} n^{C_2}_{-1\uparrow\bullet}$<br>$+n^{C_1}_{0} n^{C_2}_{0\uparrow-1\uparrow\bullet}$	1<br>2	$+n^{G_1}_{\bullet} n^{G_2}_{[-1(00)]}$<br>$+n^{G_2}_{\bullet} n^{G_1}_{[-1(00)]}$
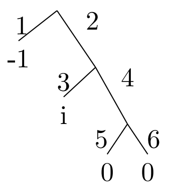	1<br>2<br>3<br>4<br>5, 6	$+n^{C_1}_{-1} n^{C_2}_{(\bullet(00))}$<br>$+n^{C_1}_{[\bullet(00)]} n^{C_2}_{-1}$<br>$+n^{C_1}_{\bullet} n^{C_2}_{(00)\uparrow-1}$<br>$+\binom{n_0^{C_1}}{2} n^{C_2}_{\bullet\uparrow-1}$<br>$+n^{C_1}_{0} n^{C_2}_{0\uparrow\bullet\uparrow-1}$	1<br>2	$+n^{G_1}_{-1} n^{G_2}_{[\bullet(00)]}$<br>$+n^{G_2}_{-1} n^{G_1}_{[\bullet(00)]}$
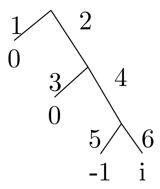	1<br>2<br>3<br>4<br>5<br>6	$+n^{C_1}_{0} n^{C_2}_{(0(-1\bullet))}$<br>$+n^{C_1}_{[0(-1\bullet)]} n^{C_2}_{0}$<br>$+n^{C_1}_{0} n^{C_2}_{(\bullet-1)\uparrow0}$<br>$+n^{c_1}_{\bullet} n^{C_1}_{-1} n^{C_2}_{0\uparrow0}$<br>$+n^{C_1}_{-1} n^{C_2}_{\bullet\uparrow0\uparrow0}$<br>$+n^{C_1}_{\bullet} n^{C_2}_{-1\uparrow0\uparrow0}$	1<br>2	$+n^{G_1}_{0} n^{G_2}_{[0(-1\bullet)]}$<br>$+n^{G_2}_{0} n^{G_1}_{[0(-1\bullet)]}$

Table S2: **Equations for computing the HDT counter ρ^X_{comb} for component types $CC \rightarrow C$ and $GG \rightarrow G$ (Cont.)** Each row shows one rooted topology of the unrooted quartet $((0,0),(-1,i))$. ρ^X_{comb} for each component type is the sum over the equations of all the rooted topologies in Table S1 and S2.

Rooted Quartet		$CC \to C$		$GG \to G$
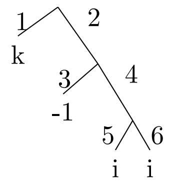	1<br>2<br>3<br>4<br>5, 6	$\sum_{i\neq j} n_i^{C_1}(n^{C_2}_{(-1(\bullet\bullet))} - n^{C_2}_{(-1(ii))} - n^{C_2}_{(-1(jj))})$<br>$+\sum_{i\neq j} n^{C_1}_{[-1(ii)]}(n^{C_2}_{\bullet} - n^{C_2}_i - n^{C_2}_j)$<br>$+n^{C_1}_{-1}(n^{C_2}_{(\bullet\bullet)\uparrow\square} - n^{C_2}_{(jj)\uparrow\bullet} - n^{C_2}_{(\bullet\bullet)\uparrow j})$<br>$+\sum_{i\neq j} \binom{n_i^{C_1}}{2}(n^{C_2}_{-1\uparrow\bullet} - n^{C_2}_{-1\uparrow i} - n^{C_2}_{-1\uparrow j})$<br>$+\sum_{i\neq j} n_i^{C_1}(n^{C_2}_{i\uparrow-1\uparrow\bullet} - n^{C_2}_{i\uparrow-1\uparrow j})$	1<br>2	$\sum_{i\neq j} n^{G_2}_{[-1(ii)]}(n^{G_1}_{\bullet} - n^{G_1}_i - n^{G_1}_j)$<br>$+\sum_{i\neq j} n^{G_1}_{[-1(ii)]}(n^{G_2}_{\bullet} - n^{G_2}_i - n^{G_2}_j)$
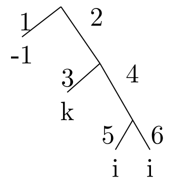	1<br>2<br>3<br>4<br>5, 6	$+n^{C_1}_{-1}(n^{C_2}_{(\bullet(\square\square))} - n^{C_2}_{(j(\bullet\bullet))} - n^{C_2}_{(\bullet(jj))})$<br>$+(n^{C_1}_{[\bullet(\square\square)]} - n^{C_1}_{[j(\bullet\bullet)]} - n^{C_1}_{[\bullet(jj)]})n^{C_2}_{-1}$<br>$+\sum_{i\neq j} n_i^{C_1}(n^{C_2}_{(\bullet\bullet)\uparrow-1} - n^{C_2}_{(ii)\uparrow-1} - n^{C_2}_{(jj)\uparrow-1})$<br>$+\sum_{i\neq j} \binom{n_i^{C_1}}{2}(n^{C_2}_{\bullet\uparrow-1} - n^{C_2}_{i\uparrow-1} - n^{C_2}_{j\uparrow-1})$<br>$+\sum_{i\neq j} n_i^{C_1}(n^{C_2}_{i\uparrow\bullet\uparrow-1} - n^{C_2}_{i\uparrow j\uparrow-1})$	1<br>2	$+n^{G_1}_{-1}(n^{G_2}_{[\bullet(\square\square)]} - n^{G_2}_{[j(\bullet\bullet)]} - n^{G_2}_{[\bullet(jj)]})$<br>$+n^{G_2}_{-1}(n^{G_1}_{[\bullet(\square\square)]} - n^{G_1}_{[j(\bullet\bullet)]} - n^{G_1}_{[\bullet(jj)]})$
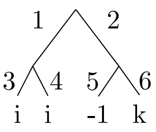	1<br>2<br>3, 4<br>5<br>6	$+\sum_{i\neq j} \binom{n_i^{C_1}}{2}(n^{C_2}_{(-1\bullet)} - n^{C_2}_{(-1i)} - n^{C_2}_{(-1j)})$<br>$+\sum_{i\neq j} n_i^{C_1} n^{C_1}_{-1}(n^{C_2}_{(\bullet\bullet)} - n^{C_2}_{(ii)} - n^{C_2}_{(jj)})$<br>$+\sum_{i\neq j} n_i^{C_1}(n^{C_2}_{i\uparrow(-1\bullet)} - n^{C_2}_{i\uparrow(-1j)})$<br>$+n^{C_1}_{-1}(n^{C_2}_{\bullet\uparrow(\square\square)} - n^{C_2}_{j\uparrow(\bullet\bullet)} - n^{C_2}_{\bullet\uparrow(jj)})$<br>$+\sum_{i\neq j} n_i^{C_1}(n^{C_2}_{-1\uparrow(\bullet\bullet)} - n^{C_2}_{-1\uparrow(ii)} - n^{C_2}_{-1\uparrow(jj)})$	1<br>2	$+\sum_{i\neq j} n^{G_1}_{(ii)}[n^{G_2}_{(-1\bullet)} - n^{G_2}_{(-1i)} - n^{G_2}_{(-1j)}]$<br>$+\sum_{i\neq j} n^{G_2}_{(ii)}[n^{G_1}_{(-1\bullet)} - n^{G_1}_{(-1i)} - n^{G_1}_{(-1j)}]$
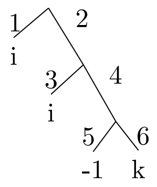	1<br>2<br>3<br>4<br>5<br>6	$+\sum_{i\neq j} n_i^{C_1}(n^{C_2}_{(i(-1\bullet))} - n^{C_2}_{(i(-1j))})$<br>$+\sum_{i\neq j} (n^{C_1}_{[i(-1\bullet)]} - n^{C_1}_{[i(-1j)]})n_i^{C_2}$<br>$+\sum_{i\neq j} n_i^{C_1}(n^{C_2}_{(\bullet-1)\uparrow i} - n^{C_2}_{(j-1)\uparrow i})$<br>$+\sum_{i\neq j} n_i^{C_1} n^{C_1}_{-1}(n^{C_2}_{\bullet\uparrow\bullet} - n^{C_2}_{j\uparrow j} - n^{C_2}_{i\uparrow i})$<br>$+n^{C_1}_{-1}(n^{C_2}_{\bullet\uparrow\square\uparrow\square} - n^{C_2}_{j\uparrow\bullet\uparrow\bullet} - n^{C_2}_{\bullet\uparrow j\uparrow j})$<br>$+\sum_{i\neq j} n_i^{C_1}(n^{C_2}_{-1\uparrow\bullet\uparrow\bullet} - n^{C_2}_{-1\uparrow i\uparrow i} - n^{C_2}_{-1\uparrow j\uparrow j})$	1<br>2	$+\sum_{i\neq j} n_i^{G_1}(n^{G_2}_{[i(-1\bullet)]} - n^{G_2}_{[i(-1j)]})$<br>$+\sum_{i\neq j} n_i^{G_2}(n^{G_1}_{[i(-1\bullet)]} - n^{G_1}_{[i(-1j)]})$

Table S3: **Equations for computing the HDT counter $\pi^X_{comb_j}$ for component types $CC \to C$ and $GG \to G$** Each row shows one rooted topology of the unrooted quartet $((i,i),(-1,k))$. $\pi^X_{comb_j}$ for each component type is the sum over the equations of all the rooted topologies.

Table S4: **E2 Results.** Comparing the running time of Q-SPR and ASTRAL-III in minutes. For the Q-SPR search method, the running time of building the starting tree is also considered. Both the heuristic and random versions of Q-SPR search are included.

Genes	ILS-Level	Runtime (minutes)			
		ASTRAL-III	Q-SPR (random)	Q-SPR (heuristic)	starting tree
$k = 50$	High	1.28	59.68	50.23	5.22
	Med	0.23	41.00	29.97	2.68
	Low	0.17	25.75	25.9	2.52
$k = 200$	High	4.37	179.72	146.52	11.12
	Med	0.48	151.77	107.97	9.98
	Low	0.27	93.82	98.60	10.02

2 Supplementary Figures

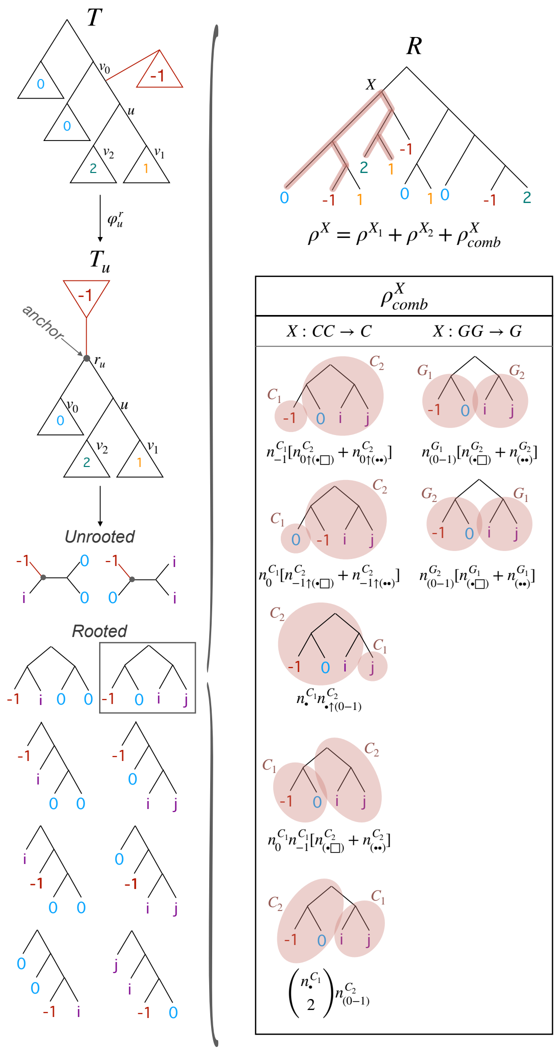

Figure S1: A detailed example of how the counter φ_u^r is computed for a node u in the query tree. The computations of ρ^X_{comb} demonstrated in this figure correspond to the equations in the first row of Table S1.

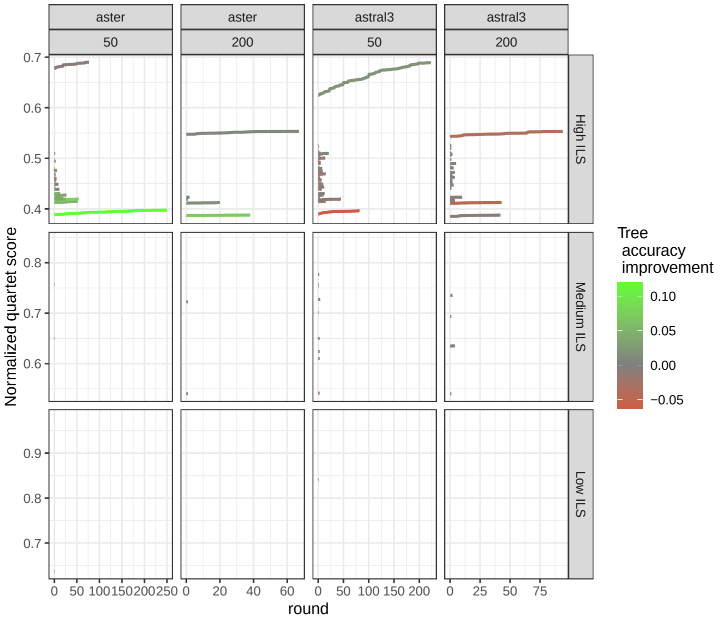

Figure S2: **E4 Results: increase in quartet score across runs.** The normalized quartet score between the Q-SPR tree at the end of each SPR round and the gene trees for all model conditions. The final improvement of the Q-SPR tree with respect to the true species tree is shown in colors.

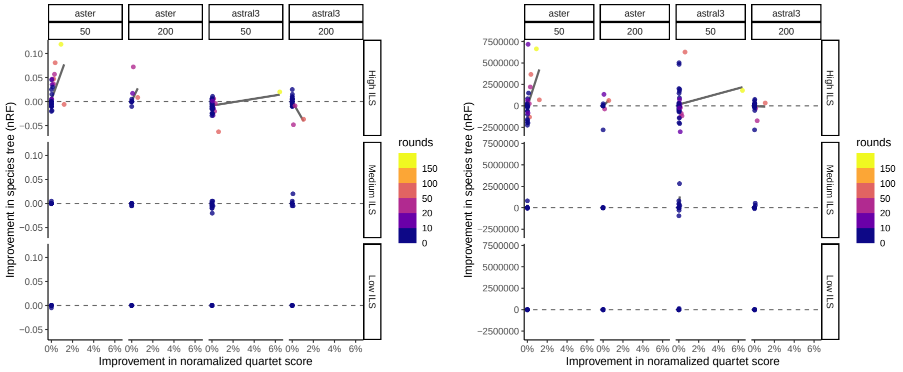

Figure S3: **E4 Results: optimization score versus accuracy.** Improvement in the quartet score of the Q-SPR algorithm above the ASTER and ASTRAL-III tree with respect to the gene trees versus the improvement in the normalized RF or the quartet distance between the ASTER tree and the true species tree. The number of SPR rounds performed for each replicate is shown in colors.

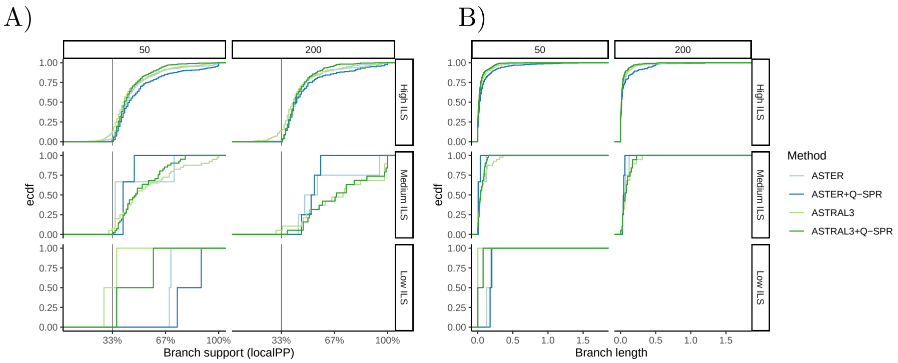

Figure S4: **E4 Results: improvements in branch support and branch length.** The results of the analysis on the non-matching branch lengths and branch supports of the improved tree using Q-SPR and the starting ASTER/ASTRAL-III tree. A) The branch supports for the improved tree. The non-matching branches refer to the branches that are either removed from the starting tree or added to the improved tree. The branch support threshold $1/3$ is shown by the dotted horizontal line. B) The empirical cumulative density function (ECDF) of the branch lengths of the improved and the starting tree for the non-matching branches.
